# Epizootiology of African Swine Fever in the Croatian Wild Boar Population and the Estimation of the Surviving Dynamics (2023–2024)

**DOI:** 10.3390/v18010015

**Published:** 2025-12-22

**Authors:** Magda Kamber Taslaman, Jelena Prpić, Margarita Božiković, Marica Lolić, Ljubo Barbić, Carmina Gallardo, Raquel Nieto, Lorena Jemeršić

**Affiliations:** 1 Laboratory for Diagnostics of Classical Swine Fever, Molecular Virology and Genetics, Virology Department, Croatian Veterinary Institute, Savska cesta 143, 10000 Zagreb, Croatia; bozikovic@veinst.hr (M.B.); jemersic@veinst.hr (L.J.); 2 Laboratory for Diagnostics, Veterinary Department Vinkovci, Josipa Kozarca 24, 32100 Vinkovci, Croatia; lolic@veinst.hr; 3 Department of Microbiology and Infectious Diseases with Clinic, Faculty of Veterinary Medicine, University of Zagreb, 10000 Zagreb, Croatia; ljbarbic@vef.unizg.hr; 4 European Union Reference Laboratory for ASF (EURL-ASF), Centro de Investigación en Sanidad Animal (CISA-INIA/CSIC), 28130 Madrid, Spain; gallardo@inia.csic.es (C.G.); nieto.raquel@inia.csic.es (R.N.)

**Keywords:** African swine fever, wild boar, qPCR, immunoperoxidase test, genetic typing, Croatia

## Abstract

This study integrates data on the prevalence, infection dynamics and risks associated with African swine fever virus (ASFV) outbreaks in Croatian wild boar during 2023–2024. Although the overall ASFV DNA prevalence in Croatia was 0.24%, the highest prevalence (2.29% in 2023 and 4.69% in 2024) was recorded in Vukovar-Srijem County. Genetic typing identified ASFV genotype II, subgroup 19, consistent with strains isolated from domestic pigs in Croatia and circulating in neighboring countries. Anti-ASFV specific antibodies were detected in 10.34% of wild boar tested in counties with previously reported DNA findings. In Vukovar-Srijem County, 4.60% of wild boar were positive for both, ASFV DNA and antibodies, suggesting ongoing virus infection, whereas the proportion of boar positive only for antibodies was 5.75%, indicating survival of acute infection. Statistical analysis revealed an increase in ASFV DNA detection from 2023 to 2024 (*p* = 0.043), with a higher prevalence in carcasses than in hunted animals (*p* = 0.001), highlighting the need for passive monitoring. While gender showed no statistical significance, a higher infection rate was observed in older animals (*p* = 0.001). The identified course of infection involved spillover events between domestic pigs and wild boar, with a significant anthropogenic influence.

## 1. Introduction

African swine fever (ASF) is a predominantly fatal viral disease of members of the *Suidae* family that causes significant socio-economic losses in affected regions. The causative agent is a large (~200 nm) DNA virus, the only member of the family *Asfarviridae*, genus *Asfivirus* [1]. To date, 24 main viral genotypes have been identified, all present in swine from Africa [2]. ASF has been spreading within sub-Saharan Africa since 1921, however, its transcontinental spread was recorded in 1957, when genotype I reached Europe, Cuba and South America. The current panzootic originated in East Africa and is caused by genotype II. The transcontinental outbreak began in 2007 in the Caucasus region (Georgia) [3] and spread primarily via wild boar across Europe, entered the European Union in 2014 and later reached Asia, Oceania and the Americas, demonstrating its presence on five continents [3,4].

In Africa, wild suids such as the African warthog (*Phacochoerus africanus*) and the giant forest hog (*Hylochoerus meinertzhageni*) act as natural virus reservoirs, showing minimal clinical signs and limited horizontal transmission [5]. In contrast, Eurasian wild boar (*Sus scrofa*) are highly susceptible, often developing acute, fatal infections similar to domestic pigs [6,7,8,9]. Nonetheless, some wild boar can survive the acute phase, enter a convalescent phase and potentially contribute to virus maintenance in the environment, a phenomenon referred to as the “wild boar–habitat” cycle, which is crucial for understanding the epizootiology of ASF in Europe [10,11]. Clinical outcomes of ASF vary according to viral virulence, infectious dose and route of infection, with mortality in peracute or acute forms approaching 100%, while subacute or mild courses result in 0–60% mortality [12,13]. Virus excretion in recovered animals may last up to six weeks or longer, supporting local virus circulation without constituting chronic infection [12,13].

Croatia’s diverse landscape, increasing wild boar density [14] and proximity to ASF-affected countries, including Hungary (2018), Serbia (2019), Italy (2022) and Bosnia and Herzegovina (2023), created favorable conditions for ASF introduction and spread [15,16,17]. To mitigate ASF risk, preventive control measures, including active and passive surveillance, have been implemented since 2019. However, the first ASF outbreak in domestic pigs was reported in June 2023, followed by the first wild boar case in July, indicating interspecies transmission and highlighting the need for coordinated control measures [18]. Despite these efforts, ASFV persisted in the wild boar population, raising questions about their role as reservoirs and contributors to environmental viral persistence.

The aim of this study is therefore to gain a better undestanding of ASF outbreaks in the Croatian wild boar population through integrated active and passive surveillance and environmental testing over a two-year period. The results of this research will expand our understanding of viral spread and the role of wild boar as potential virus carriers by detecting both acutely infected and convalescent animals, and will also reveal the virulence characteristics of the ASFV strain circulating in Croatia under natural conditions. The results should specify targeted wildlife management and improve ASF control strategies, ultimately contributing to enhanced biosecurity in domestic pig production.

## 2. Materials and Methods

The research is carried out at the Laboratory for Classical Swine Fever Diagnostics (CSF), Molecular Virology and Genetics of the Croatian Veterinary Institute (CVI) in Zagreb, which is accredited according to the HRN EN ISO/IEC 17025:2007 standard and has been appointed by the Ministry of Agriculture (MoA) as the National Reference Laboratory of the Republic of Croatia for CSF and ASF diagnostics. Testing was also conducted at the Official Laboratory for ASF in the Vinkovci Department of CVI (since August 2024). Additionally, genetic typing was performed at the European Union Reference Laboratory (EURL) for ASF (CISA-INIA/CSIC, Madrid, Spain).

As part of the ASF active and passive surveillance programs in Croatia, established from 2019 by MoA, based on the Regulation on Control Measures for the Control of ASF in Croatia (OG 147/23), Regulation 2016/429, Delegated Regulation 2020/687, Implementing Regulation 2023/594 and the National Animal Health Act (OG 152/22, 154/22), samples for the detection of ASF were collected by hunters from the hunting grounds of twenty one Croatian counties.

### 2.1. Samples Included in This Study

In 2023 and 2024, a total of 21,934 wild boar samples (sera, spleen and bone marrow) were collected as part of the active and passive surveillance programs supported by MoA. All samples were taken and sent by official hunters and were tested for the detection of ASFV DNA. In addition, 174 samples were selected for serological testing, focusing on counties with confirmed qPCR-positive cases to investigate ASFV survival and seroprevalence in wild boar following outbreaks. The number of samples is based on a 95% confidence level within the total population of 21,934 wild boar.

Samples were categorized on the basis of active (regularly hunted wild boar with no signs of disease) and passive surveillance (wild boar carcasses and remains) and classified into age groups of up to six months, six months to one year, one to two years and over two years old, on the basis of tooth eruption and/or replacement. Both male and female wild boar were represented in the data set (Table 1).

In addition, five soil and two fecal samples were collected in Vukovar-Srijem County in the Spačva hunting ground (45°03′38.1″ N 18°53′21.5″ E), due to reports of wild boar movement and carcasses found in the area.

### 2.2. ASFV DNA Extraction and Real-Time Polymerase Chain Reaction

Prior to analysis, sera samples were prepared by centrifugation at 1500 rpm/10 min and stored at 4 °C to 8 °C until testing. Spleen and bone marrow samples were prepared by manual homogenization (1 g of tissue resuspended in 10 mL of sterile phosphate buffered saline, PBS) vortexed for one minute and centrifuged at 3000 rpm for five minutes, then stored at −20 °C until testing. Soil and fecal samples were collected (1–5 g) and PBS was added in a ratio of 1:10 in sterile Falcon tubes. Before testing, samples were mixed using vortex for one minute and centrifuged at 3000 rpm for ten minutes. They were stored at −20 °C until testing.

DNA was extracted by an automated isolation procedure performed by a King Fisher Flex device (Thermo Fisher Scientific, Waltham, MA, USA) using the IndiMag Pathogen Kit (Indical Bioscience GmbH, Leipzig, Germany) according to the manufacturer’s instructions. The exogenous positive control (Non-Target Positive Control, NTPC-ASF) was added to monitor the presence of potential PCR inhibitors. In addition, a negative ASF wild boar serum sample was included in the extraction procedure to ensure that there was no cross-contamination. A weak positive reference standard positive serum (RSPS) of wild boar prepared as a validation parameter was also included in the protocol. Isolates that were not immediately subjected to real-time polymerase chain reaction (qPCR) were stored at −20 °C until use.

QPCR was performed by the CFX96 instrument (Biorad, Hercules, CA, USA) using ID Gene™ African Swine Fever Triplex Kit (ID vet, Grabels, France) according to the manufacturer’s instructions. Each reaction included an internal positive control (IPC), i.e., a positive amplification control (PAC-ASF), RSPS and a negative control. A fluorescent signal with a cycle threshold (Ct) value below 35 was considered positive. This cutoff is established based on the kit’s producer’s validation, ensuring reliable detection of ASFV genetic material. The Universal Probe Library (UPL) method, which is recommended by the EURL for ASF and described in the World Organization for Animal Health (WOAH) manual [19], was performed to confirm positive ID Gene ASF Triplex results. The procedure was performed using the ORA™ qPCR Probe Mix 2X (HighQu GmbH, Kraichtal, Germany), targeting an ASFV DNA fragment within the VP72 coding genomic region [20]. According to the guidelines provided by the EURL (CISA-INIA, Madrid, Spain) for ASF diagnostics and WOAH, a fluorescent signal with a Ct value below 40 was considered positive. This cut-off value is set based on the validated performance parameters of the assay to ensure that the amplification signal corresponds to the presence of ASFV DNA and not to background noise or non-specific reactions. A Ct value below 40 indicates that sufficient viral genomic material is present in the sample to allow reliable detection within the defined sensitivity of the method.

### 2.3. Indirect Immunoperoxidase Test

ASFV-specific antibodies were detected using an indirect immunoperoxidase test (IPT) validated by the EURL for ASF and performed according to its standard operating procedure (SOP) [21]. Sera, spleen and bone marrow samples were tested using antigen-coated plates containing fixed, ASFV-infected VERO cells provided by the EURL.

### 2.4. Genetic Typing

Twelve representative samples, eight from domestic pigs and four from wild boar, were selected for genotyping. Initial screening was performed by partial Sanger sequencing of a fragment of the B646L/p72 gene as previously described [22]. Further molecular characterization followed the multi-gene approach described by Gallardo et al. [2], targeting six genomic regions (CVR within B602L, IGR I73R/I329L, complete O174L, partial K145R, MGF 505-9R/505-10R, and ECO2 I329L/I215L). PCRs were performed with published primers using AmpliTaq Gold DNA Polymerase (Thermo Fisher Scientific, Waltham, MA, USA). Amplicons were purified with VWR^®^ ExoCleanUp FAST (VWR International GmbH, Darmstadt, Germany), sequenced bidirectionally by Sanger sequencing with the BrilliantDye v1.1 Kit (NimaGen BV, Nijmegen, Netherlands), purified with the Optima DTR™ 96-Well Plate kit (Qiagen, Hilden, Germany) and analyzed on a 3730 DNA Analyzer (Applied Biosystems, Foster City, CA, USA). Sequence quality was checked with Chromas (www.technelysium.com.au, accessed on 13 October 2025), aligned in MEGA v11 software [23], using the ClustalW algorithm with adjusted gap penalties to preserve epidemiologically informative indels (pairwise gap opening = 3, extension = 0.1; multiple alignment gap opening = 5, extension = 0.2). Following inspection and trimming of each alignment to homologous length, the six region-specific alignments were concatenated into a single composite alignment, producing one concatenated sequence per isolate.

The final concatenated alignment included 85 sequences, comprising the twelve new isolates and at least two representative isolates per genetic subgroup and/or country to capture diversity of genotype II. Phylogenetic reconstruction was performed in MEGA v11 using the Maximum Likelihood (ML) method under the General Time Reversible (GTR + G) model, selected by model testing. Bootstrap resampling (1000 replicates) was applied to assess node support. Gaps and missing data were handled by partial deletion with a 95% site-coverage cutoff, thus retaining informative indel positions.

All nucleotide sequences generated in this study were deposited in the EURL ASF sequence databank (available at https://asf-referencelab.info/sequence-database-info/, accessed on 13 October 2025).

### 2.5. Statistical Analysis

Non-parametric Chi-square tests (Pearson’s Chi-square and Goodness of Fit) were performed to assess associations between ASF prevalence in 2023 and 2024 and to compare prevalence rates between carcasses and hunted wild boar, gender and age groups with 95% prevalence confidence intervals (CI). Additionally, odds ratios (OR) with 95% CI were calculated using logistic regression to quantify the association between ASF infection and categorical variables such as geographical distribution, gender and age groups. A *p*-value < 0.05 was considered statistically significant.

## 3. Results

### 3.1. Detection of ASFV-DNA by qPCR

The overall national prevalence of ASFV DNA positive cases was 0.15% in 2023 and increased to 0.30% in 2024 on the basis of 21,934 samples tested.

In 2023, 13 cases of ASFV DNA positive wild boar were detected in Croatia. The first case was confirmed on the 5 July 2023 in hunting ground XVI/108 Mašanj in Vukovar-Srijem County. In total, there were nine positive wild boar in Vukovar-Srijem, two in Sisak-Moslavina, one in Zadar and one in Karlovac County.

In 2024, 39 wild boar samples tested positive, 38 of which were from hunting ground XVI/11 Spačva in Vukovar-Srijem County, approximately one kilometre from the locations of outbreaks in domestic pigs and near the borders with Serbia and Bosnia and Herzegovina (up to 20 km). In November 2024, there was an additional case of ASF in hunting ground XVI/129 Vučedol, also in Vukovar-Srijem County.

ASFV DNA positive cases in wild boar are presented in Table 2 according to year of detection, location and prevalence by county.

**Figure 1:**
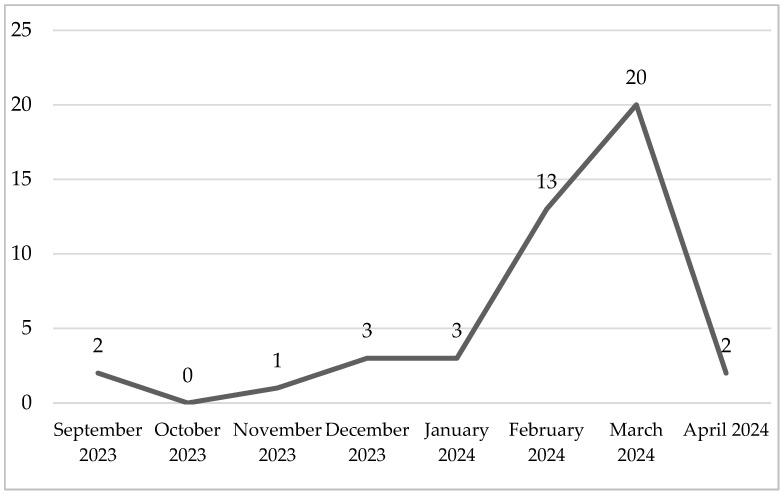
ASFV DNA positive cases in Spačva hunting ground in 2023 and 2024 according to the number of positive samples per month.

In 2023, in Vukovar-Srijem County the prevalence was 2.29%, and it increased to 4.69% in 2024. The highest number of ASF-positive cases was observed in the Spačva hunting ground in Vukovar-Srijem County. The outbreak in this region peaked in March 2024, with 20 reported positive cases, followed by a sharp decline in April (Figure 1).

None of the environmental soil and feces samples were positive for ASFV DNA.

### 3.2. Detection of Anti-ASFV Antibodies by IPT

IPT was performed on 36 sera, 93 spleen and 45 bone marrow samples. Seropositive cases are presented by location and prevalence by county in Table 3.

**Figure 2:**
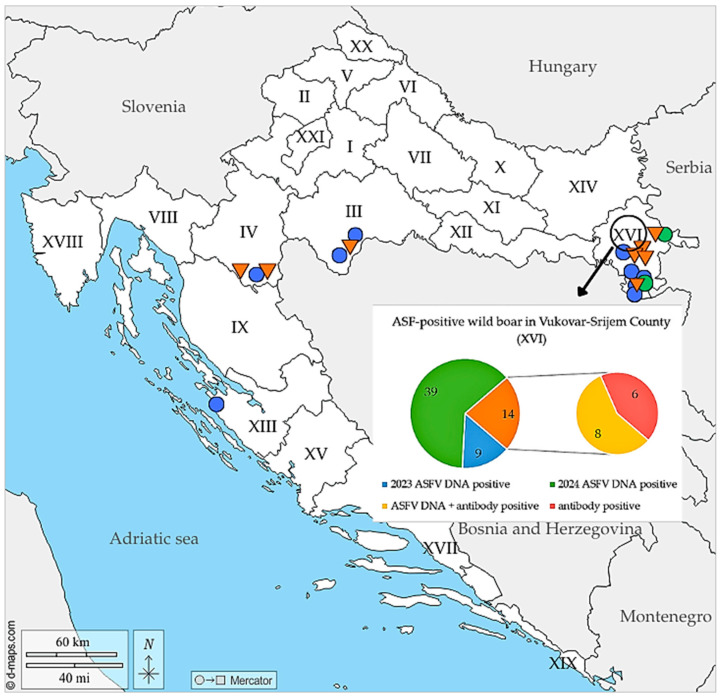
Distribution of ASF infection stages in wild boar across affected Croatian counties during 2023 and 2024.

Figure 2 presents the geographical distribution of seropositive wild boar cases together with ASFV DNA-positive detections recorded in 2023 and 2024.

Roman numerals present Croatian counties: Bjelovar-Bilogora (VII), Brod-Posavina (XII), Dubrovnik-Neretva (XIX), Istria (XVIII), Karlovac (IV), Koprivnica-Križevci (VI), Krapina-Zagorje (II), Lika-Senj (IX), Međimurje (XX), Osijek-Baranja (XIV), Požega-Slavonia (XI), Primorje-Gorski kotar (VIII), Sisak-Moslavina (III), Split-Dalmatia (XVII), Šibenik-Knin (XV), Varaždin (V), Virovitica-Podravina (X), Vukovar-Srijem (XVI), Zadar (XIII), Zagreb (I) and the City of Zagreb (XXI).

Blue circles indicate hunting grounds with ASFV DNA-positive wild boar detected in 2023, while green circles indicate ASFV-DNA positive findings in 2024. Orange triangles denote hunting grounds where seropositive wild boar were identified. The inset highlights Vukovar-Srijem County (XVI), the most affected region, and summarizes infection stages: numbers of animals testing ASFV-DNA positive in 2023 and 2024, seropositive only, and those positive for both ASFV-DNA and antibodies.

Table 4 summarizes the infection stage of wild boar based on the detection of ASFV DNA and anti-ASFV antibodies.

**Figure 3:**
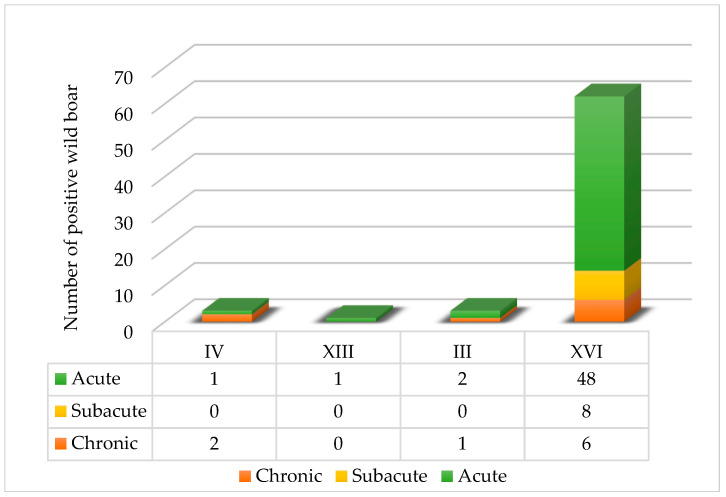
Distribution of ASF infection stages in wild boar population across Croatian counties.

As shown in Figure 3, the distribution of ASF infection stages among wild boar varied geographically, with acute, subacute and chronic infections occurring differently across counties.

The majority of ASFV-positive wild boar were classified as acutely infected, particularly in Vukovar-Srijem County (XVI), where 48 acutely infected cases were recorded. Subacute infections were exclusively found in Vukovar-Srijem, with eight cases, while chronic infections were observed across three counties: Vukovar-Srijem (XVI, six cases), Karlovac (IV, two cases) and Sisak-Moslavina (III, one case). Notably, no subacute or chronic infections were detected in Zadar County (XIII).

### 3.3. Genetic Characterization of ASFV Isolates Using a Multigene Approach

A total of 16 ASFV-positive samples were analyzed, including 12 from domestic pigs and 4 from wild boar, collected in Croatia between June 2023 and July 2024 from different outbreak locations (Table 5). Samples were selected to represent host and temporal diversity while ensuring sufficient viral load for sequencing.

Twelve ASF partial B646L/p72 sequences were aligned with reference strains of ASFV genotypes I–XXIV. All Croatian samples clustered within genotype II and showed very high nucleotide identity (99.7–100%) with sequences of genotype II previously reported from Eurasia.

Molecular characterization followed the multigene approach proposed by Gallardo et al. [13], targeting six genomic regions (CVR within B602L, IGR _I73R/I329L_, complete O174L, partial K145R, intergenic region MGF _505-9R/505-10R_, and ECO2 _I329L/I215L_). All samples showed 100% identity across the six loci sequenced. The CVR region corresponded to variant I with 10 amino acid TRS, identical to the Georgia 2007/1 strain (FR682468.2). The IGR locus was consistently variant II, the most frequent subtype described in over 92% of Eurasian strains [24]. No variation was detected in O174L and K145R, both classified as variant I. Analysis of the MGF region revealed two sets of tandem repeats, ABBCD in the first block and EFGHH in the second, resulting in a total of 11 TRS. This configuration corresponds to MGF variant I and was identical to Georgia 2007/1 [2,24]. Finally, all isolates grouped with ECO2 variant II, defined by a SNP in the I215L gene leading to an amino acid substitution (Glu192Gly) as previously described [2].

**Figure 4:**
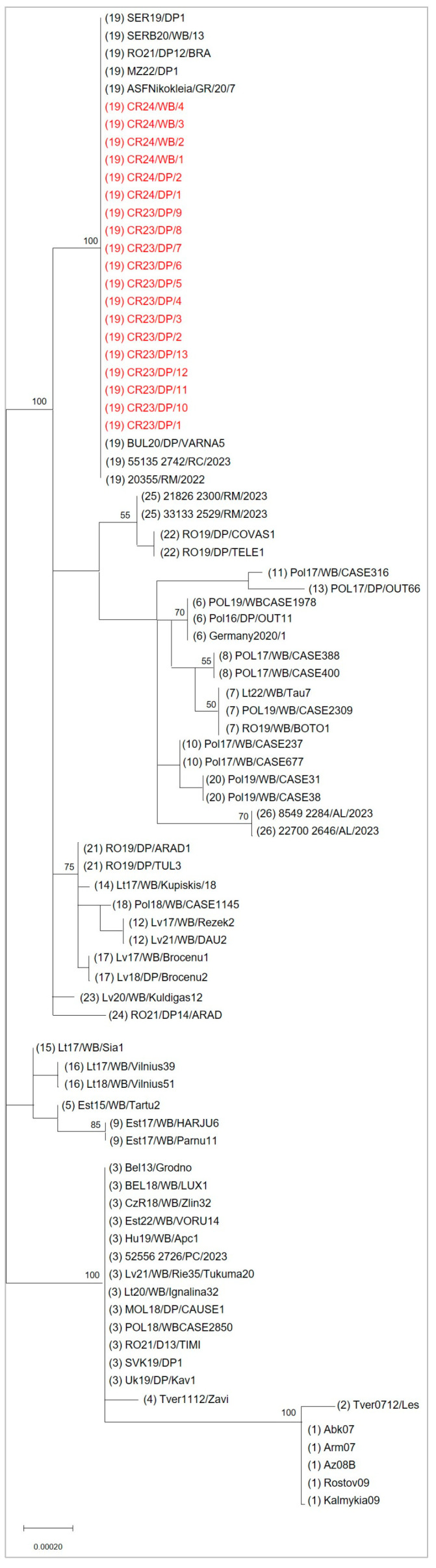
Phylogenetic tree of ASFV genotype II isolates based on concatenated nucleotide sequences of six variable genomic regions (CVR, IGR, O174L, K145R, MGF 505-9R/505-10R, and ECO2). The evolutionary history was inferred using the Maximum Likelihood method under the GTR + G model. The tree is drawn to scale, with branch lengths representing the number of substitutions per site. Only bootstrap values > 50% (from 1000 replicates) are shown next to the branches. The dataset included 85 concatenated sequences and positions with gaps or missing data were treated by partial deletion (95% cutoff). There were a total of 2405 positions in the final dataset. Evolutionary analyses were conducted in MEGA11. Croatian isolates are shown in red, and the genetic subgroup assigned to each strain is indicated in parentheses after the isolate name.

The combination of these markers (CVR-I, IGR-II, O174L-I, K145R-I, MGF-I, ECO2-II) allowed the assignment of all Croatian isolates to genetic subgroup 19, according to the classification of Gallardo et al. [2]. Table 5 summarizes the ASFV-positive samples, specifying isolate ID, host species, location, sampling date and genetic profiles based on multiple markers (CVR, IGR, O174L, K145R, MGF, ECO2), as well as their assigned genetic subgroup. Phylogenetic analysis of the concatenated alignment (85 sequences, including references) corroborated this assignment (Figure 4): Croatian sequences formed a single, well-supported cluster (bootstrap = 100) together with contemporary genotype II isolates from Serbia, Greece, Bulgaria, Romania, Italy and North Macedonia [2,24].

### 3.4. Statistical Findings

Using the Pearson Chi-Square test, a statistically significant difference was found in the number of ASFV DNA-positive wild boar cases between 2023 and 2024 (*p* = 0.043), confirming an increasing trend in the detection of infection. ASFV DNA was detected significantly more frequently in wild boar carcasses compared to hunted animals (*p* = 0.001). Seasonality was also evaluated as a potential risk factor for ASFV DNA detection, with a significantly higher prevalence observed in winter compared to summer (OR = 13.89; 95% CI: 5.23–36.89; *p* = 0.00000011).

A statistically significant difference between age groups was detected using the Chi-Square Goodness-of-Fit test (*p* = 0.001), with the highest prevalence found in animals older than one year and the lowest in those younger than six months. No statistically significant difference was found in ASFV incidence between male and female wild boar (*p* = 0.6242). Likewise, there was no significant difference in the proportion of seropositive animals between carcasses and hunted wild boar (*p* = 0.6315), indicating comparable seroprevalence in both groups. However, the age-stratified analysis of seropositive animals revealed a significant difference (*p* = 0.001), with higher seropositivity in older individuals. No significant gender-specific differences were found among seropositive wild boar (*p* = 0.8065).

Karlovac County (IV) was selected as the reference group for odds ratio (OR) calculations due to its exceptionally low ASF prevalence (one positive case among 1786 wild boar tested; 0.06%), providing a stable baseline for comparison. By comparing ORs in Sisak-Moslavina (III), Vukovar-Srijem (XVI) and Zadar (XIII) Counties with Karlovac, the relative risk associated with geographical location was quantified. Males were the reference for gender-specific comparisons and the youngest age group (<6 months) was the reference for age-related analysis. This approach enabled evaluation of changes in infection probability with increasing age (Table 6 and Table 7).

Counties XVI and XIII showed a markedly higher ASF prevalence compared to the reference County IV. These counties exhibited very high odds ratios (72.90 and 17.16, respectively) with statistically significant *p*-values (<0.001 and 0.045), indicating a strong geographical influence on ASF occurrence. Gender was not significantly associated with ASF detection (*p* = 0.200). ASF prevalence increased with age, though none of the age-related ORs were statistically significant.

No statistically significant correlation was found for any examined risk factors. County XVI had the highest seroprevalence (20%), followed by County IV (11.11%) and County III (2.17%). The OR for County XVI is 2.00 (95% CI: 0.52–7.74), suggesting a possible trend, but the wide confidence interval and high *p*-value limit confidence in this effect. Gender and age groups showed small differences in seroprevalence, but none reached statistical significance.

## 4. Discussion

This study provides the first comprehensive evidence that ASF in Croatian wild boar exhibits acute, subacute and past infection courses. Active viral circulation was documented in Vukovar-Srijem County, with notable subacute cases of 4.60%. Serological evidence of past exposure was detected in 5.75% of wild boar in Vukovar-Srijem, Sisak-Moslavina and Karlovac Counties.

Following the first ASF outbreak in domestic pigs in Posavski Podgajci (Vukovar-Srijem County) on the 23 June 2023, the first ASF-positive wild boar was detected shortly afterwards on the 5 July 2023 in Mašanj, approximately 10 km from the affected farm. In total, during 2023, Croatia reported 1124 ASF outbreaks in domestic pigs, mostly in small-scale holdings (<100 pigs) with low biosecurity measures. The close temporal and spatial association between positive pigs and the first cases of ASF in wild boar highlights the possibility of virus spillover into the wild boar population. Inadequate fencing and proximity to wild boar habitats have certainly facilitated virus transmission. While alternative anthropogenic pathways, such as illegal hunting and improper carcass disposal, cannot be excluded, the regional epizootiological context supports domestic-to-wild boar transmission as a plausible contributor to these first wild boar cases, contrasting with the typical European pattern in which wild boar usually represent the primary source of infection [10]. Besides Vukovar-Srijem County, sporadic ASFV-positive wild boar cases were detected in 2023 in Sisak-Moslavina, Zadar and Karlovac counties, though these cases had minimal impact on the overall disease spread.

In 2024, the number of outbreaks in domestic pigs dropped markedly to six, reflecting successful containment measures. In neighboring Serbia, 310 outbreaks were recorded in 2024, also mainly in small-scale holdings, while other bordering countries reported fewer cases [25]. These data indicate that ASFV circulation in domestic pigs persisted in the region, maintaining the potential for further transmission, although at a reduced level. However, the situation changed in 2024 when the highest number of ASF-positive cases was detected in the Spačva hunting ground in Vukovar-Srijem County. Spačva is an oak forest and ecologically rich lowland covering over 25,000 hectares, some of which border Serbia and Bosnia and Herzegovina. It proved to be the outbreak hotspot, with 38 out of 39 ASFV DNA-positive wild boar cases, detected from January to the end of April 2024. The temporal peak of the outbreak was reached in March, followed by a significant decline in April. The statistically significant increase in ASFV DNA positive cases in 2024 compared to 2023 (*p* = 0.043) highlights the intensification of virus circulation within the wild boar population over time.

This study also highlights the possibility that wild boar can survive acute infection, rather than succumb to it. In Vukovar-Srijem County, 4.60% of tested wild boar were positive for both ASFV DNA and anti-ASFV antibodies, indicating prolonged viral presence and potential infectiousness beyond previously reported durations [12]. These animals may contribute to virus maintenance and spread even without visible symptoms, emphasizing the importance of active surveillance programs. Serological testing also revealed seropositive wild boar in three counties (Vukovar-Srijem, Karlovac and Sisak-Moslavina) but no detectable ASFV DNA, indicating past exposure and recovery. The presence of seropositive animals in areas without prior outbreaks, such as Sisak-Moslavina and Karlovac, suggests medium-risk zones and indicate possible silent virus circulation. These findings underscore the need for integrated surveillance combining active and passive approaches to detect both, currently infected and previously exposed wild boar, ensuring a more comprehensive understanding of ASF epizootiology.

The flooding of the Sava River in August 2023, following heavy rains, likely facilitated ASFV spread in Vukovar-Srijem County by reducing the effectiveness of containment measures and physically moving wild boar carcasses, some of which were observed floating in the floodwaters. To evaluate the potential for environmental transmission, soil and fecal samples were collected in October 2024, six months after the Spačva outbreak, at sites where ASFV-positive carcasses had previously been found. All samples tested negative for ASFV DNA, suggesting that the environment was unlikely to serve as a source of infection. However, the number of collected samples was limited due to restricted access to additional sites after control measures were implemented. Environmental persistence of ASFV is influenced by temperature, UV radiation and microbial activity, which generally promote viral inactivation [26]. Nevertheless, in November 2024, a new ASF case was detected in the Vučedol hunting ground, approximately 50 km from the Spačva outbreak. These findings suggest that local environmental sources were not responsible for this new case; instead, the virus was likely introduced through the movement of recently infected or convalescent wild boar. In contrast to environmentally related pathways, human-mediated factors appear to play a more significant role in sustaining ASF transmission in Croatia, especially among domestic pigs, but also indirectly in wild boar. Surveys of hunting practices and farm management indicate that these pathways are particularly relevant in Vukovar-Srijem County and neighboring regions.

Phylogenetic analysis of the B646L/p72 gene confirmed that all Croatian ASFV isolates, regardless of their origin, collected between June 2023 and July 2024 belong to genotype II, with nucleotide identities exceeding 99.7% when compared to reference strains. The application of the multigene approach [2] (CVR, IGR, O174L, K145R, MGF and ECO2) further demonstrated that all 16 samples analyzed were genetically homogeneous, showing complete identity across the six loci examined. Based on the updated classification of ASFV genotype II viruses circulating in Europe, which now distinguishes 28 genetic subgroups [2], all Croatian isolates were assigned to genetic subgroup 19. Unlike the situation reported in Italy, where several genetic subgroups and novel variants (e.g., subgroup 25 and 26) have been identified, the Croatian dataset did not reveal any new marker patterns, single nucleotide polymorphisms (SNPs), or alternative tandem repeat sequence (TRS) structures. This homogeneity supports a relatively recent introduction of ASFV into Croatia, with limited time for genetic diversification. The detection of subgroup 19 in Croatia is consistent with its wide distribution in Southeastern and Eastern Europe and is predominant in Romania, Bulgaria, Serbia, Greece, and North Macedonia [2] as well as in Italy, where it has been associated with both wild boar and domestic pig outbreaks [24]. The finding that all Croatian isolates, from both wild boar and domestic pigs, belong to subgroup 19 indicates that the outbreak is part of the broader regional circulation of this lineage, without evidence of multiple introductions or diversification events, to date.

Consistent with observations from other European ASF affected countries such as Poland [27], Estonia [9] and Lithuania [28,29], this study demonstrated that ASFV DNA positive wild boar in Croatia were detected significantly more frequently among animals found dead than among those hunted, showing a statistically significant difference (*p* = 0.001). In 2023, 61.8% of ASF-positive detections originated from carcasses, a proportion that increased to 95% in 2024. This finding reinforces the epizootiological value of passive surveillance, which is more likely to detect animals that have died from acute ASF infection and therefore provides a more accurate reflection of active virus circulation. In contrast, active surveillance is the prefered tool when targeting surviving animals and potential reservoirs of the virus. Moreover, active surveillance can contribute to the recognition of disease or re-introduction of the virus before its manifestation or after the recognition of an outbreak. This is imperative and of crucial importance in combating ASF. Hence, integrating robust passive surveillance and active surveillance into ASF monitoring strategies can ensure effective outbreak detection and improve control interventions.

The seasonal pattern of ASF cases observed in our study is consistent with findings from other European countries, including Bulgaria, Estonia, Germany, Latvia, Lithuania and Romania [30]. Our data indicate a significantly higher transmission rate in the winter months, while in summer, new ASF cases in wild boar occur sporadically and are primarily the result of spillover events from infected domestic pigs. Lower environmental temperatures in winter slow down the decomposition of wild boar carcasses, thereby prolonging the viability of ASFV in the environment and increasing the risk of indirect transmission through contact with infectious remains [31,32,33].

Geographical location was a significant risk factor for ASFV DNA positivity in Croatian wild boar, with Vukovar-Srijem County showing the highest prevalence (3.92%) and infection probability (*p* < 0.0001) compared to Karlovac, while Zadar County also had an elevated risk. However, small sample sizes and wide confidence intervals warrant caution. Age trends suggested higher ASFV positivity in older animals, likely reflecting cumulative exposure and wider habitat use, but logistic regression *p*-values did not confirm independent age effects. This pattern aligns with findings from Lithuania [28,29] but contrasts with Estonia [34], where younger animals were more affected. Ecological factors such as predation of piglets may contribute to underrepresentation of the youngest age group. No significant differences were observed between genders, indicating that gender is not a major determinant of ASFV exposure, although males showed a slightly higher seroprevalence rate.

Although this study addressed important gaps in understanding ASF epizootiology, several limitations should be considered. First, the detection rate of ASFV DNA may have been affected by under-detection of carcasses in densely forested habitats, where limited visibility and difficult access reduce the likelihood of recovering samples. Second, the serological analysis was based on a relatively small subset of samples (*n* = 174), limiting statistical power and the generalizability of seroprevalence estimates. The samples were selected as representative according to their quality (absence of degradation), origin, sampling location, gender and age, as most (95%) were collected from wild boar carcasses found in counties with confirmed qPCR-positive cases. Third, the assessment of environmental transmission was limited. Sampling was restricted to soil and fecal material at a few accessible sites, precluding a comprehensive evaluation of ASFV persistence in the environment. Additionally, the role of scavengers in virus transmission and maintenance remains poorly understood, as this study did not include systematic observations of their presence or activity, particularly in the case of jackals that may share habitats with wild boar in Croatia. Fourth, small sample sizes in certain strata, particularly County XIII (*n* = 105) and younger age groups, may have limited the statistical power of the analyses, as indicated by wide confidence intervals and the inability to detect significant associations in logistic regression models. Although the elevated odds ratio for County XIII suggests a potentially increased risk, the wide confidence interval indicates considerable uncertainty. These results should therefore be interpreted with caution, and future studies with larger sample sizes are needed to provide more robust risk estimates.

Finally, long-term ecological monitoring through longitudinal studies is essential to assess how ASFV persists and spreads. However, our data make a considerable contribution to a better understanding of the epizootiology of ASF in the wild boar population.

## 5. Conclusions

This study reveals the epizootiological characteristics of ASF among the wild boar population in Croatia, with Vukovar-Srijem County identified as a primary hotspot marked by ongoing ASFV circulation. The detection of ASFV in Vukovar-Srijem County in both 2023 and 2024, along with evidence of subacute and post-infection stages, including seropositive cases in Karlovac and Sisak-Moslavina Counties, suggests local viral persistence and the potential role of surviving wild boar in maintaining viral spread. These findings highlight the importance of continued, integrated passive and active surveillance, particularly in high-risk areas such as the Spačva hunting ground in Vukovar-Srijem County, where indications of local endemicity are emerging. Sustained molecular and serological monitoring, together with coordinated regional control measures, will be essential to mitigate the ongoing risk of spillback from wild boar populations to domestic pigs. Given the demonstrated spillover and spillback circulation of ASFV among domestic pigs and wild boar, the potential for the development of local endemicity of ASF, particularly in North East Croatia, is a pressing epizootiological risk.

## Figures and Tables

**Table 1:** Samples collected as a part of the ASF surveillance programs in Croatia in 2023 and 2024.

Category	Subcategory	2023	2024
status	H	8359 (95.55%)	9202 (69.79%)
	C	390 (4.46%)	3983 (30.21%)
age group	<6 months	595 (6.80%)	1151 (8.73%)
	6 months–1 year	2643 (30.21%)	3472 (26.33%)
	1–2 years	2662 (30.43%)	3678 (27.89%)
	>2 years	2479 (28.34%)	4144 (31.44%)
	n.d.	370 (4.22%)	740 (5.61%)
gender	M	3998 (45.70%)	6129 (46.48%)
	F	4624 (52.85%)	6771 (51.36%)
	n.d.	127 (1.45%)	285 (2.16%)

Abbreviations: H—hunting sample; C—carcass sample; n.d.—not determined; F—female; M—male.

**Table 2:** Prevalence of ASFV DNA positive cases in wild boar in 2023 and 2024 with 95% Confidence Intervals (CI).

County (Code)	Hunting Grounds (Coordinates)	Total Samples	Positive	Negative	Prevalence (95% CI)
2023					
Karlovac (IV)	XXII/168 Rakovica (44°59′35.72″ N 15°39′10.23″ E)	1786	1	1785	0.06% (0.01–0.32%)
Sisak-Moslavina (III)	III/121 Staza (45°22′36.0″ N 16°35′48.5″ E), III/40 Zrinska Gora II (45°10′00.0″ N 16°15′18.5″ E)	3527	2	3525	0.06% (0.02–0.21%)
Vukovar-Srijem (XVI)	XVI/102 Drenova (44°57′04.8″ N 18°53′00.7″ E), XVI/108 Mašanj (44°52′56.9″ N 18°49′05.7″ E), XVI/114 Selište (44°59′01.3″ N 18°58′28.0″ E), XVI/11 Spačva (45°03′38.1″ N 18°53′21.5″ E), XVI/14 Trizlovi-Rastovo (45°08′08.0″ N 18°47′06.6″ E)	393	9	384	2.29% (1.06–4.27%)
Zadar (XIII)	XIII/115 Blatski gaj (44°09′05.4″ N 15°15′01.0″ E)	105	1	104	0.95% (0.02–5.19%)
Σ	-	5811	13	5798	0.22% (0.13–0.38%)
2024					
Σ Vukovar-Srijem (XVI)	XVI/11 Spačva (45°03′38.1″ N 18°53′21.5″ E), XVI/129 Vučedol (45°20′21.3″ N 19°02′13.8″ E)	831	39	792	4.69% (3.45–6.35%)

**Table 3:** Seropositive cases in wild boar with 95% confidence intervals (CI).

County (Code)	Hunting Grounds (Coordinates)	Total Samples	Positive	Negative	Prevalence (95% CI)
Karlovac (IV)	IV/117 Tušilović (45°20′46.05″ N 15°32′33.70″ E)	26	2	24	7.69% (0.94–25.13%)
Sisak-Moslavina (III)	III/127 Petrinja (45°24′48.3″ N 16°20′45.6″ E)	47	1	46	2.13% (0.05–11.21%)
Vukovar-Srijem (XVI)	XVI/147 Aljmaš (45°30′59.3″ N 18°56′26.1″ E), XVI/11 Spačva (45°03′38.1″ N 18°53′21.5″ E), XVI/16 Vrapčana (45°15′49.4″ N 18°50′13.1″ E), XVI/129 Vučedol (45°20′21.3″ N 19°02′13.8″ E)	69	15	54	21.74% (12.94–33.04%)
Zadar (XIII)	-	32	0	32	0%
Σ	-	174	18	156	10.34% (6.09–15.82%)

**Table 4:** Stage of infection and proportion of ASF positive wild boar based on the detection of ASFV DNA and anti-ASFV specific antibodies.

Detected	Infection Stage	Positive/Tested Samples (Proportion %)
ASFV DNA	acute infection	52/21,934 (0.24%)
ASFV DNA + anti-ASFV antibodies	subacute infection	8/174 (4.60%)
anti-ASFV antibodies	chronic infection	10/174 (5.75%)

**Table 5:** ASFV-positive samples analyzed by multigene sequencing in Croatia (2023–2024).

Isolate	Host	Location	Date	CVR	IGR	O174L	K145R	MGF	ECO2	Genetic Subgroup
CR23/DP/1	DP	Posavski Podgajci	23 June 2023	I	II	I	I	I	II	19
CR23/DP/2	DP	Posavski Podgajci	23 June 2023	I	II	I	I	I	II	19
CR23/DP/3	DP	Posavski Podgajci	23 June 2023	I	II	I	I	I	II	19
CR23/DP/4	DP	Posavski Podgajci	23 June 2023	I	II	I	I	I	II	19
CR23/DP/5	DP	Posavski Podgajci	23 June 2023	I	II	I	I	I	II	19
CR23/DP/6	DP	Posavski Podgajci	23 June 2023	I	II	I	I	I	II	19
CR23/DP/7	DP	Moživić	7 October 2023	I	II	I	I	I	II	19
CR23/DP/8	DP	Moživić	8 October 2023	I	II	I	I	I	II	19
CR23/DP/9	DP	Moživić	8 October 2023	I	II	I	I	I	II	19
CR23/DP/10	DP	Semeljci	9 October 2023	I	II	I	I	I	II	19
CR23/DP/11	DP	Moživić	10 October 2023	I	II	I	I	I	II	19
CR23/DP/12	DP	Vrbica	10 October 2023	I	II	I	I	I	II	19
CR23/DP/13	DP	Kupina	16 October 2023	I	II	I	I	I	II	19
CR24/WB/1	EWB	Spačva, Vrbanja	19 January 2024	I	II	I	I	I	II	19
CR24/WB/2	EWB	Spačva, Vrbanja	21 February 2024	I	II	I	I	I	II	19
CR24/WB/3	EWB	Spačva, Vrbanja	6 March 2024	I	II	I	I	I	II	19
CR24/WB/4	EWB	Spačva, Vrbanja	6 March 2024	I	II	I	I	I	II	19
CR24/DP/1	DP	Gradište	8 July 2024	I	II	I	I	I	II	19
CR24/DP/2	DP	Gradište	10 July 2024	I	II	I	I	I	II	19

Abbreviations: CR—Croatian; DP—domestic pig; EWB—European wild boar.

**Table 6:** Analysis of risk factors associated with ASFV DNA positivity in Croatian wild boar.

Risk Factor	Variable	Examined Wild Boar (N)	Prevalence (%)	OR ^1^	95% CI ^2^	*p*-Value
County	IV	1786	0.06	reference		
	III	3527	0.06	1.01	0.09–11.19	0.994
	XVI	1224	3.92	72.90 *	10.10–522.70	<0.001 *
	XIII	105	0.95	17.16 *	1.07–276.20	0.045 *
Gender	M	2055	1.17	reference		
	F	4587	0.57	2.07	1.19–3.61	0.200
Age	<6 months	746	0.06	reference		
	6 months–1 year	1258	0.72	5.37	0.37–22.33	0.320
	1–2 years	2232	0.85	6.41	0.66–37.29	0.120
	>2 years	1906	1.05	7.92	0.64–35.70	0.130

^1^ odds ratio, ^2^ confidence interval for OR, * statistically significant *p* < 0.05.

**Table 7:** Analysis of risk factors associated with ASF seropositivity in Croatian wild boar.

Risk Factor	Variable	Examined Wild Boar (N)	Prevalence (%)	OR ^1^	95% CI ^2^	*p*-Value
County	IV	27	11.11	reference		
	III	46	2.17	0.18	0.02–1.70	0.140
	XVI	70	20	2.00	0.52–7.74	0.284
Gender	M	60	13.3	reference		
	F	83	10.84	1.27	0.54–3.88	0.215
Age	<6 months	17	5.88	reference		
	6 months–1 year	25	4	0.67	0.02–18.92	0.495
	1–2 years	45	13.3	2.46	0.28–21.93	0.184
	>2 years	56	16.07	3.06	0.37–25.48	0.124

^1^ odds ratio, ^2^ confidence interval for OR.

## Data Availability

The authors declare that the data supporting the findings of this study are available within the article. Additional information is available from the authors upon reasonable request.

## Abbreviations

The following abbreviations are used in this manuscript:

Abbreviation	Definition
ASF	African Swine Fever
ASFV	African Swine Fever Virus
qPCR	Real-time Polymerase Chain Reaction
IPT	Immunoperoxidase Test
CSF	Classical Swine Fever
CVI	Croatian Veterinary Institute
MoA	Ministry of Agriculture
PBS	Phosphate Buffered Saline
rpm	Revolutions per Minute
Ct	Cycle Threshold
IPC	Internal Positive Control
PAC-ASF	Positive Amplification Control for ASF
RSPS	Reference Standard Positive Serum
UPL	Universal Probe Library
SOP	Standard Operating Procedure
WOAH	World Organisation for Animal Health
EURL-ASF	European Union Reference Laboratory for African Swine Fever
SNPs	single nucleotide polymorphisms
TRS	tandem repeat sequence
